# Proteomic Insights into the Biology of the Most Important Foodborne Parasites in Europe

**DOI:** 10.3390/foods9101403

**Published:** 2020-10-03

**Authors:** Robert Stryiński, Elżbieta Łopieńska-Biernat, Mónica Carrera

**Affiliations:** 1 Department of Biochemistry, Faculty of Biology and Biotechnology, University of Warmia and Mazury in Olsztyn, 10-719 Olsztyn, Poland; ela.lopienska@uwm.edu.pl; 2 Department of Food Technology, Marine Research Institute (IIM), Spanish National Research Council (CSIC), 36-208 Vigo, Spain

**Keywords:** foodborne parasite, food, proteomics, biomarker, liquid chromatography-tandem mass spectrometry (LC-MS/MS)

## Abstract

Foodborne parasitoses compared with bacterial and viral-caused diseases seem to be neglected, and their unrecognition is a serious issue. Parasitic diseases transmitted by food are currently becoming more common. Constantly changing eating habits, new culinary trends, and easier access to food make foodborne parasites’ transmission effortless, and the increase in the diagnosis of foodborne parasitic diseases in noted worldwide. This work presents the applications of numerous proteomic methods into the studies on foodborne parasites and their possible use in targeted diagnostics. Potential directions for the future are also provided.

## 1. Introduction

Foodborne parasites (FBPs) are becoming recognized as serious pathogens that are considered neglect in relation to bacteria and viruses that can be transmitted by food [1]. The mode of infection is usually by eating the host of the parasite as human food. Many of these organisms are spread through food products like uncooked fish and mollusks; raw meat; raw vegetables or fresh water plants contaminated with human or animal excrement. Most FBPs are related to outdated farming procedures and/or to wild animals [2]. Some food is contaminated by food service employees who do not follow sanitation rules or work in unsanitary facilities. The globalization of food supply, the increase of international trade, the convenience of travel, the increase of highly susceptible people (such as aging, malnutrition, human immunodeficiency virus infection), changes in cooking traditions and lifestyles, and advanced diagnostic tools are some of the reasons for the increase in the incidence of food-borne parasitic diseases worldwide [3,4,5].

In a general context, it was confirmed that there might be up to 50% more species benefit from parasitic lifestyle than all other feeding strategies [6]. Parasites, in particular protozoa (Protozoa), roundworms (Nematoda), flukes (Trematoda), and tapeworms (Cestoda) are enormously different types of eukaryotes that may cause human infection. Their complex lifecycles; varied transmission routes, including water, soil, food, and contacts between people or between animals and people; as well as prolonged periods between infection and symptoms have resulted in their receiving considerable attention in the last few decades [3,7]. The most examined species, *Homo sapiens,* can serve as host to 342 different helminth species and to 70 more if we count the Protozoa. According to local geographic, ecological and economic conditions, every human population in the world has its own unique suit of parasites [8]. The development of animal husbandry, sanitary conditions and diagnostic methods has undoubtedly reduced or even eliminated certain parasite species in industrialized countries and some developing countries. However, the decrease in the number of cases is not common for all parasitic species, especially for foodborne parasites, and there are countries where the occurrence of these infections in humans is still high [2].

Monitoring of foodborne diseases is a fundamental component of food-safety systems. The European Union has introduced regulations for some FBPs, such *Trichinella* spp.*, Taenia* spp. and *Anisakis* spp. [9,10,11]. There are currently no European Union standards published exclusively for Protozoa in food products. However, after massive cryptosporidiosis outbreaks, the food industry began to pay attention to *Cryptosporidium* spp. [12,13,14]. Validated methods are essential to ensure robust detection of FBPs. The current guidelines are used to monitor bacteria and their direct application to FBPs is not possible. Other concerns include the large differences in FBPs populations (from protozoa to parasitic worms) and their biological differences (for example, different transmission routes, complex development cycles). The detection procedures of FBPs in food products are also different. There are also differences in the range of foods that FBPs may exist and be delivered to potential human hosts. Because of the complex development cycle of many FBPs and the wide variety of hosts, the food analyzed for the detection of a specific FBP can include uncooked meat, fish and other seafood, and fruits or vegetables. [15]. In these cases, research is needed to identify new, more specific treatment targets.

In recent years, proteomics methods have become more and more popular in the food science community [16,17,18,19]. New methods for detecting parasites are still an urgent research matter that can successfully benefit from proteomic methodologies.

Proteomics is defined as “the large-scale functional analysis of gene products or functional genomics, including identification or localization studies of proteins” [20]. Proteomics methods are used for the identification and quantification of the protein composition of cells, subfractions of cells, or the medium or secretome surrounding cells at a certain time, collectively termed “the proteome,” but also to describe protein modifications and interactions [17]. Proteomic analysis involves the extraction, purification and fractionation of proteins which are identified using mass spectrometry (MS) [21]. Earlier proteomic studies generally used two-dimensional gel electrophoresis (2-DE) approach separating protein mixtures according to charge (pI) and molecular weight (MW), after which proteins could be identified using MS [22]. Today, bottom-up or “shotgun” proteomic approaches that analyze proteins after proteolytic digestion can be coupled to liquid chromatography tandem mass spectrometry (LC-MS/MS), which allows for the high-throughput quantitation of the proteome [23]. These require a high quality and representative genome sequence to map thousands of MS spectra of peptides back to their proteins for identification and quantitation and allow for the characterization of whole proteomes. These conventional approaches were not possible to use in parasitological studies until now. These days, the full or extensive nuclear genome coverage for many parasites of agricultural, veterinary, and medical importance [24] makes proteomics more interesting for parasitologists.

In order to confirm the identity of novel proteins and their function, the application of proteomics is a key to enable extensive characterizations of them or, more widely, the structures and organelles where those proteins are expressed and which are essential in pathogenicity but which are in many cases lacking in more widely studied model organisms, for instance yeast or *Caenorhabditis elegans*–free-living nematode [25]. To fully understand the molecular mechanisms related to the pathogenic characteristics of FBPs, it is most important to analyze the surface proteins and the proteins in extracellular vesicles that represent the frontier of interaction between the parasite and its host. [26,27,28,29]. In the past decade, people have used the latest technological advances in proteomics and bioinformatics to develop proteomics strategies to analyze this complex class of molecules [19]. The identification of parasite-specific proteins can significantly simplify the design of new tools for fast and inexpensive diagnosis, which in turn can help break the spread of parasites. In addition, identifying potential vaccination targets (proteins) appears to be one of the leading ways to control parasitic diseases [30]. Accurate knowledge and description of the mechanism of action of these proteins can be used in the research on antiparasitic drugs, and help in combating FBPs through detection and/or neutralization [31]. At the same time, scientists are making great efforts to clarify the sensitization mechanism of various allergenic proteins from food sources, where allergic reactions to food are more often caused by FBP allergens contaminating food products [3,32,33].

**Figure 1:**
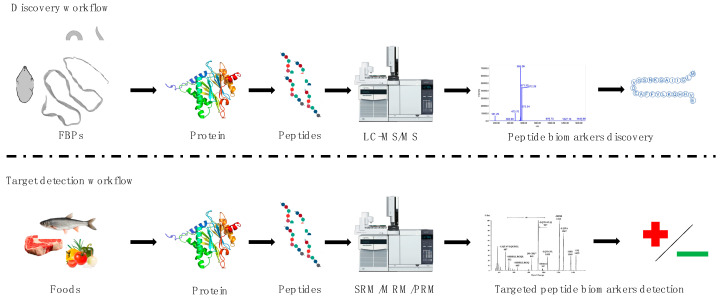
Schematic representation of two proteomic strategies in the studies of foodborne parasites (FBPs): discovery and targeted workflows. Red plus—positive detection (sample contaminated); green minus—negative detection (sample free); LC-MS/MS—liquid chromatography tandem mass spectrometry; SRM/MRM/PRM—selected/multiple/parallel—reaction monitoring.

Detection of specific parasites in humans and the etiological cause of the disease, like food products, required scientists to employ two characteristic approaches using proteomic methods, i.e., discovery and targeted workflows [17,18,34]. Discovery proteomics is applied to identify and characterize the proteins of FBPs (e.g., global proteomes, cellular, subcellular, or excretory-secretory proteomes) usually employing previously mentioned, bottom-up methodology. Targeted proteomics are based on the monitoring of the protein biomarkers (single or multiple peptides) in analyzed samples, e.g., food products. In targeted proteomics, selected/multiple/parallel-reaction monitoring (SRM/MRM/PRM) is preferably used [35] (Figure 1).

The aim of this paper is to present a review of the proteomics methods applied to (i) discovery phase —the studies of FBPs with particular attention to identifying and characterizing new targets for treatment and diagnosis, and to (ii) targeted detection phase —selected FBPs detection in food products.

## 2. Discovery Approach—Description of the Selected FBPs and the Proteomics Methods Used to Study Them

Priorities in FBP differ at the global and European levels. In this work, we focused on the most important emerging FBPs in Europe according to the ranking for risk management of FBPs created for the recommendation of the Food and Agriculture Organization of the United Nations (FAO) and the World Health Organization (WHO) [36]. Additionally, we divided FBPs according to transmission routes (Table 1). An overview on the systematics of selected parasites is provided in Table 2. A brief description of selected FBPs with emphasis on proteomics methods used to study them is provided below. For more detailed information on the pathogenesis and surveillance of diseases caused by foodborne parasites, please see the individual disease health topic pages and factsheets on the European Centre for Disease Prevention and Control website (https://www.ecdc.europa.eu/en) and additionally on the Centers for Disease Control and Prevention website (https://www.cdc.gov) and the World Health Organization website (https://www.who.int).

### 2.1. Waterborne Parasitic Species

Water is the main habitat for many lifestages of parasites. These stages can contaminate food products or directly infect humans via the drinking of infected water. Among waterborne parasitic infection, according to the European ranking [36], the most important parasites are *Cryptosporidium* spp. (fifth/25), *Giardia lamblia* (eight/25), *Entamoeba histolytica* (14th/25), *Cyclospora cayetanensis* (21st/25), and *Spirometra* spp. (25nd/25).

#### 2.1.1. *Cryptosporidium* spp.

*Cryptosporidium* spp. is one of the main causes of human diarrhoeal diseases and, with *Giardia lamblia*, is a major cause of protozoan waterborne diseases [3,37]. In addition to diarrhea, the general symptoms associated with cryptosporidiosis include nausea, vomiting, loss of appetite, and cramps. Cryptosporidiosis is having a clear link with impaired cognitive and functional development in children in developing countries [38]. The lifecycle of *Cryptosporidium* spp. is completed within a single host. In immune-competent individuals, the symptoms of cryptosporidiosis are often self-limiting, but may be chronic when the immune system is compromised, such as in children under 5 years of age or cancer patients [39]. There is currently no effective medication for cryptosporidiosis [40], and so far, nitazoxanide is the only drug accepted by U.S. Food and Drug Administration. Despite that, as mentioned earlier, it is still ineffective, most of all for immunosupressive patients, such as people living with HIV [41]. Between 1984 and 2017, waterborne disease outbreaks, as well as infections through unpasteurized milk and dairy products, and through handling animals, and infections by using recreational waters were caused by 25 outbreaks of cryptosporidiosis [3,7,37].

The use of proteomics techniques was important during the studies of *Cryptosporidium* spp. Sanderson et al. [42] conducted an extensive analysis of the proteome of excysted *C. parvum* sporozoites. Three independent proteomics methods were used to maximize the coverage of the proteome: (i) 2-DE LC-MS/MS; (ii) 1-DE LC-MS/MS; and (iii) multi-dimensional protein identification technology (MudPIT) analysis, in which trypsin-digested peptides were separated by multi-dimensional LC and then subjected to MS/MS. Over than 4800 protein spectra have been identified. These proteins represent 1237 non-redundant proteins, what is one third of the entire proteome of *C. parvum.* For example, Siddiki and Wastling [43], used mass spectrometry-based basic local alignment search tool (MS BLAST) to identify *C. parvum* proteins from frozen sporozoite pellets isolated from lamb feces. They separated the total protein by one-dimensional sodium dodecyl sulphate–polyacrylamide gel electrophoresis (1D-SDS-PAGE) and analyzed by two-dimensional nano-liquid chromatography-tandem mass spectrometry (2D-n-LC MS/MS). Using this method, the authors found 84 proteins specific for *C. parvum*, one third of which were previously hypothetical. In another study, Snelling et al. [44] also used MS, but they not only tried to analyze the proteome of Cryptosporidium, but also aimed to determine proteins that were differentially regulated in the excysted sporozoites compared to the non-excysted. Their proteomic analysis shown the expression of 26 proteins, which were significantly modulated after excystation. Interestingly, 3 of the proteins were specific for apicomplexan, and 5 were specific for Cryptosporidium. The authors proposed that, all identified proteins may be involved in pathogenesis. However, it remains to be determined whether Cryptosporidium causes the same response when it comes into contact with the host or is internalized. Beside these “omic” studies, using an in silico approach, the novel drug target (protein) was described and characterized using predicted proteome and bioinformatics methods. However, an in vitro or in vivo study is needed to confirm the above proposition [45].

At present, we hardly understand which proteins interact with the parasite/host, because the data retrieved from the host-parasite protein-protein interaction from the Cryptosporidium-infected hosts is very limited.

#### 2.1.2. *Giardia lamblia*

*Giardia lamblia* (*G. lamblia*) (also known as *Giardia intestinalis* and *Giardia duodenalis*) is a single-celled protozoan parasite that can infect the small intestines of humans and animals. Giardiasis occurs globally across socioeconomic boundaries but is mainly endemic in developing countries and particularly within young children [22]. Main symptoms of giardiasis are diarrhea, epigastric pain, nausea, vomiting and weight loss, and they appear 6–15 days after infection and are more severe on young children and individuals with malnutrition or immunodeficiency. Giardiasis is usually treated with metronidazole or other nitroimidazoles [46]. 

Proteomics were widely used in the studies on *G. intestinalis*. Originally, the secretome of *G. intestinalis* after in vitro co-incubation with human intestine cell lines (Caco-2 cells or HT-29) was analyzed using a 2D gel-based approach, with proteins identified using matrix-assisted laser desorption ionization time-of-flight mass spectrometry (MALDI-TOF MS) [47]. This experiment identified three metabolic *G. intestinalis* proteins (arginine deiminase, ornithine carbamoyl transferase, and enolase) and two human proteins (enolase and HSP70). Then, the Giardia secretome was further and more deeply explored in subsequent studies [48,49]. Ma’ayeh et al. [49] analyzed the *Giardia* secretome during host–parasite interaction between differentiated Caco-2 cells and isolates from both *G. intestinalis* human-infective assemblage (WB and GS) using LC-MS/MS. Dubourg et al. [48] also identified steady-state, axenic-secreted proteins in *G. intestinalis* (WB and GS isolates) proteins. The label-free method was used for protein quantification; the intensity-based absolute quantification (iBAQ) approach, which calculates the sum of parent ion intensities of the peptides identified in each protein. Secreted proteins were identified based on ratios between protein abundance of proteins analyzed in whole trophozoite lysate compared to protein abundance from culture supernatants. Higher supernatant to lysate ratios of protein abundance were considered indicative of enrichment due to active parasite secretion and used to eliminate cytoplasmic contaminants derived from parasite lysis [48]. The findings from these studies supports observations that secreted proteases are important virulence factors in breaking down host gut barriers and modulating host immune responses. Recently it was demonstrated that *Giardia* trophozoites release microvesicles (MV) which play key role in proliferation and parasite-host interaction. The protein repertoire of peripheral vesicles (PVs) and encystation-specific vesicles (ESVs) has been described [50]. To describe the first protein composition of ESVs and PVs, a novel strategy combining flow cytometry-based organelle sorting with in silico filtration of mass spectrometry data was used (SDS-PAGE and LC ESI-MS/MS) [50]. The protein composition of MV was also analyzed using MS from both trophozoites and cysts, with 11 and 80 proteins identified in MV from each lifestage, respectively [51].

Recently, in order to expand the variety of vaccine candidate antigens, Davids et al. [52] considered that surface proteins can be a rich source of such antigens. In their study, the trophozoites reacted with non-membrane-permeant NHS biotin. The biotin-labeled and unlabeled controls were evaluated by immunofluorescence. Differential interference contrast microscopy was used to compare cells. All cell lysates were prepared in parallel and analyzed either directly or immunoprecipitated using streptavidin-agarose beads. The precipitated proteins were separated by SDS-PAGE, and gels were visualized by Coomassie staining. The biotinylated proteins were detected by immunoblotting with an anti-biotin antibody. Immunoprecipitants were then digested and analyzed by LC-MS/MS [52].

The latest research on the surface proteome of *G. intestinalis* used shotgun mass spectrometry to analyze the proteomes of trophozoites of the three different strains. This allowed the identification of 2368 proteins, among which, using monoclonal antibodies, the variant-specific surface proteins were identified [53].

Accordingly, the proteomics helped to conclude that there is an extensive host-parasite crosstalk during *Giardia* infections of different human cell lines via both secretome and direct interactions.

#### 2.1.3. *Cyclospora cayetanensis*

*Cyclospora cayetanensis (C. cayetanensis*) is an apicomplexan, coccidian protozoan parasite, which in 1994 was described as a causative agent of human cyclosporiasis—a self-limiting diarrheal disease, the symptoms of which are also fatigue, loss of appetite, nausea, and vomiting [54]. Infection occurs through the ingestion of contaminated water or agricultural products (raspberries, basil, and coriander) [55,56]. Since the spore formation time after fecal shedding is longer (at least seven days), the possibility of infection between people is less. Cyclosporiasis is endemic in Nepal, Peru, and Haiti [57,58].

Little is known about the proteome of *C. cayetanensis*. Cinar et al. [59] sequenced the *C. cayetanensis* genomic DNA extracted from clinical stool samples, annotated, and used the sequence as a reference for proteome prediction. Therefore, they validated the quality of such reference by comparing the predicted proteome of related parasites (including Cyclospora and Babesia). The analysis showed 29 core apicomplexan proteins found in most apicomplexans [59]. A similar method was recently used by Liu et al. [60], who proved that the *C. cayetanensis* genome may encode as many as 7457 proteins. Among them, 538 proteins had signal peptides (of which 105 target the apicoplast), 1247 had one or more transmembrane regions, and 225 had a GPI anchor attachment site. These data are similar to those in *Eimeria tenella* and *Toxoplasma gondii* [60].

#### 2.1.4. *Entamoeba histolytica*

Amoebiasis is a disease caused by the protozoan parasite *Entamoeba histolytica* (*E. histolytica*). When a person swallows something contaminated with *E. histolytica* cysts (water or food products), infection may occur [61]. Only about 10% to 20% of people who are infected with *E. histolytica* will become ill due to the infection. Symptoms include loose stools, stomach pain, and stomach cramps. Amoebic dysentery is a serious form of amoebiasis and is related to stomach pain, bloody stools and fever. *E. histolytica* rarely invades the liver and forms an abscess [62].

Most of the proteomic publications have focused on analyzing the expression profiles of trophozoites in different organelles under numerous conditions [61]. By 2-DE followed by MS, whole extracts have been analyzed, including both soluble and insoluble proteins, cytoskeletal, membrane, and signaling-associated proteins [63,64]. Other teams focused on investigating phagosome composition and proteins associated with the phagocytosis process [65], the cell surface protein profile [66], and a nuclear and cytoplasmic proteomes of trophozoites [67]. In a more recent study, the membrane proteome of *E. histolytica* was described [68], and the excretory-secretory proteins were identified [69]. The components of trophozoite ER and Golgi apparatus were characterized as well [70]. In addition, by comparing the proteome of *E. histolytica* with related non-pathogen amoeba *E. dispar* it was possible to identify proteins related to virulence and pathogenicity [71,72,73,74]. The latest studies focused not only on the trophozoite stage of *E. histolytica* but also on the cysts and cyst-like structures gained from trophozoites [61,75]. Moreover, *E. histolytica* exposed to serum isolated from a person with amoebiasis, induces cell polarization by activating signal transduction pathways and cytoskeletal components. This process results in the formation of a protruding pseudopod at the front of the cell and a retracted uropod at the rear. Marquay Markiewicz et al. [76], using LC-MS/MS, showed the proteomic composition of the uropod fractions.

The proteins identified in presented studies that may be recognized by the immune system and/or released into the circulatory system during amoebiasis, and may be a detectable biomarker of the disease.

#### 2.1.5. *Spirometra* spp.

The procercoid larvae or plerocercoid larvae of *Spirometra* tapeworms are the cause of sparganosis in humans [77]. Humans are an intermediate host for the parasite, who acquire sparganosis most often by drinking water contaminated with infected copepods (intermediate host) or consuming the meat of an undercooked second intermediate host (fish, reptiles, amphibians). Once ingested by a human, the larvae undergo visceral migration and can end up in many tissues, where they grow [78]. Depending on the final location of the parasite, migrating sparganum can cause various symptoms. It may be located in almost every part of human body, including subcutaneous tissue, breasts, orbits, urinary tract, lungs, pleural cavity, abdominal viscera and central nervous system [78]. The migration of subcutaneous tissue is commonly painless. However, when the parasite settles in the one of the elements of central nervous system, like brain or spine, a variety of neurological symptoms may occur, including weakness, headaches, seizures and abnormal skin sensations such as numbness or tingling. If the inner ear is affected, the patient may experience dizziness or may even become deaf for a short time [77].

Proteomic methods have been used to characterize *Spirometra* tapeworms. 2-DE was used to describe the protein expression differences between three different stages of *S. erinacei*, the plerocercoid larvae, eight-day-old juveniles, and adults [79]. The specific or highly expressed proteins in juvenile worms were analyzed by MALDI-TOF MS/MS. The proteome profile of larvae showed fewer protein spots than juveniles or adults, and juveniles and adults showed similar protein expression profiles. Eight juvenile-specific proteins and five juvenile up-regulated proteins were identified and their functions were determined [79]. Immunoproteomic analyses of *S. erinaceieuropaei* and *S. mansoni* have also been performed [80,81]. Both studies used 2-DE and Western blot probed with sera from infected mice. Protein spots which showed immune response were characterized by MALDI-TOF/TOF-MS. The recent study on *S. erinaceieuropaei* sparanga described site-specific phosphoproteome, with the purpose of describing the global phosphorylation status of spargana [82]. A total of 1758 spargana proteins were identified, where 3228 phosphopeptides and 3461 phosphorylation sites were described among. This dataset provides a valuable data repository for future research on the metabolic pathways of this important zoonotic parasite.

### 2.2. Soil- and Plant-Borne Parasitic Species

Infective developmental stages of parasites are spread with fecal-contaminated soil and may contaminate food products like editable aquatic plants (e.g., watercress, algae), vegetables, fruits, and fruit juices. Among soil- and plant-borne parasitic infection, according the European ranking [36], the most important parasites are *Echinococcus multiocularis* (first/25), *E. granulosus* (4th/25), *Toxocara* spp. (ninth/25), *Ascaris* spp. (12th/25), *Fasciola* spp. (17th/25), *Trypanosoma cruzi* (19th/25), and *Trichuris trichiura* (22nd/25).

#### 2.2.1. *Echinococcus multiocularis* and *E. granulosus*

Six species of genus *Echinococcus* have been described, of which two are of public health importance in Europe: *E. granulosus* (the causative agent of cystic echinococcosis—CE) and *E. multiocularis* (the causative agent of alveolar echinococcosis—AE) [36]. The infection is caused by the accidental consumption of fruit and vegetables contaminated with parasite eggs shed by a carnivore final host [3]. For both these species, humans are accidental intermediate hosts [3]. In humans, the liver is the most common site for cystic and alveolar echinococcosis [83].

In CE, cysts grow slowly (1–5 cm in diameter per year). It may take many years to show any symptoms, usually due to organ dysfunction in which the cyst grows. If a cysts ruptures, the sudden release of its contents can cause sensitization ranging from mild to severe anaphylactic shock [84]. The immunodiagnostic techniques coupled with anamnesis, and radiological imaging are used for diagnostic purposes of echinococcosis where, the steps for diagnosing AE in humans are the same as those for CE [85,86]. There are several main treatment options, including surgery, puncture aspiration injection reaspiration, and chemotherapy. For asymptomatic individuals, consideration of a “waiting and watching” approach with the supervision of the patient is recommended [83,87].

The proteomes of *E. granulosus* and *E. multiocularis* are still not well described, but there are some reports of proteomic studies on those parasites. In 2003, for the first time the proteomic analysis of *E. granulosus* protoscoleces by 2-DE and peptide mass fingerprinting (PMF) was performed [88]. Host serum proteins (especially albumin and globulin) were highly concentrated in the samples, what caused horizontal streaks on the hydatid fluid 2-DE gels. Even when parasite hydatid fluid-enriched fraction was prepared, large amounts of bovine serum albumin and globulins still made it complicated for 2-DE to detect parasite-specific proteins. A few more studies describing protoscolex proteome using 2-DE were performed, some of which were followed by MS analysis [89,90,91,92,93]. Then, Monteiro et al. [94] used LC-ESI-Q-TOF MS/MS to analyze protoscolex and the hydatid cystic fluid of *E. granulosus*. Moreover, Longuespée et al. [95] in order to describe the proteomics model of CE in the liver, used the latest laser microdissection-based proteomics and MALDI-MS workflow. This study demonstrated specific markers of a parasitic cyst in the liver. The comparison of *E. multilocularis* and *E. granulosus* hydatid fluid protein composition was also done using LC-ESI-MS/MS and provides explanation of specialized host–parasite interactions [96]. The proteome of an adult stage of *E. granulosus* was also described [97]. Moreover, the extracellular vesicles derived from *E. granulosus* and their protein composition was also presented [98].

#### 2.2.2. *Toxocara* spp.

Toxocariasis is caused by the transmission of *Toxocara* species from carnivores (canines or cats) to humans. The most widespread and very epidemiologically important species in the world, *T. canis* can infect a large group of canines, like dogs, foxes, wolves, jackals, and coyotes, while *T. cati* can infect cats [99]. The human can accidentally ingest *Toxocara* eggs containing infectious third-stage larvae (L3) from contaminated food, environment (soil or sand) and/or water. The L3 larvae hatch the egg, and migrate through the wall of the host’s small intestine, and then can get through the circulatory system to the variety of organs, including liver, lungs, central nervous system and/or muscle tissue [100]. Most infections are asymptomatic, and since clinical investigations and/or diagnostic tests are not usually performed, human diseases may go unnoticed but sometimes larvae cause immune and inflammatory reactions, resulting in symptoms including fever, headache, cough, and pain in the abdomen or limbs. There is currently no vaccine against toxocariasis. Human chemotherapy differs depending on the symptoms and location of the larva but usually is limited to albendazole or mebendazole administered with anti-inflammatory corticosteroids [99].

Few studies using proteomics methods have been conducted on *Toxocara* spp. In this review, we focused only on describing *T. canis*. The *T. canis* genome contains at least 18,596 protein-coding genes, and their predicted products include at least 373 peptidases, 458 kinases, 408 phosphatases, 273 receptors, and 530 transporters and channels. In addition, the secretory proteome (870 molecules) of *T. canis* is rich in proteases that are probably involved in the penetration and degradation of host tissues, and rich in molecules that are recommended to inhibit the host’s immune response [101]. Analysis of excretory-secretory products and larval extract using 1D-SDS-PAGE-LC-MS/MS has been done by da Silva et al. [102], who identified 646 proteins (582 somatic and 64 excretory-secretory), among which, many may play a role in parasite-host interactions, as well as in regulating parasite metabolism and survival. A similar approach was used by Sperotto et al. [103], who identified 19 proteins. According to the classification using the signal peptide predicted by SignalP [104], 7 of the identified proteins were located outside the cell, 10 have cytoplasmic or nuclear localization, and the subcellular localization of the two remain proteins was unknown [103].

This advancement in *Toxocara* proteomics has brought hope to medicine and veterinary medicine, especially in the areas of better diagnostic tools, effective vaccines or drugs.

#### 2.2.3. *Ascaris* spp.

*Ascaris lumbricoides* (*A. lumbricoides*) and *A. suum* are infecting humans and pigs, respectively [105]. *A. lumbricoides*, as a one of the most common parasites in the world is infecting 1.2 billion people worldwide [106]. In human and pig hosts, the migration route of larvae is similar. After ingesting infectious ova, L3 larvae covered by the L2 cuticle hatch in the small intestine and migrate to the caecum and proximal colon, where they penetrate the mucosa. The larvae then migrate via the portal vein to reach the liver, where the L2 cuticle is shed. The larvae then migrate through the portal vein to reach the liver. After migrating in the liver, the larvae enter the lungs 6-8 days post infection (p.i.), then penetrate the alveolar space and move to the pharynx, where they are swallowed, what is causing larvae to return to the small intestine on day 8–10, where larvae molt to L4 development stage. Larvae mature and reach sexual maturity on day 24 p.i. and during that time is molting last (L5) [105]. Due to the hepato-tracheal migration, the infection with *A. lumbricoides* may cause pulmonary and intestinal symptoms such as persistent sore throat, dyspnoea, sometimes coughing up blood, abdominal pain, nausea, vomiting, and diarrhea. In addition, eosinophilic pneumonia (pulmonary ascariasis) or obstruction of the intestinal lumen in the case of intestinal ascariasis may occur in case of severe infection. Ascariasis is treated pharmacologically by administering albendazole or mebendazole in a single dose [107].

Despite the public health importance impact, both parasites proteomes’ are poorly described. In the latest paper, Xu et al. [108], described the use of 2-DE coupled with MALDI-TOF/TOF MS to compare proteomes of adult female *A. lumbricoides* and *A. suum*. In the six gels examined (three gels for each parasite species), more than 630 and 750 protein spots were repeatedly found. After comparing the 2-DE proteomes of *A. lumbricoides* and *A. suum*, it was found that the protein profiles of the two species were very similar, with almost no differentially modulated proteins. The protein expression profiles determined by 2-DE coupled with MALDI-TOF/TOF MS method were about three times higher than those obtained with 2-DE [109]. Analysis of only head ends of 10 immature *A. lumbricoides* and *A. suum* using MALDI-TOF MS was also performed, but only to describe protein profiles (characteristic protein peaks) of those species [110]. Most of the proteomic studies were performed on *A. suum*. Proteomic analysis of the excretory–secretory products from larval stages (L3-egg, L3-lung, and L4) of *A. suum* by LC-MS/MS revealed high abundance of glycosyl hydrolases. Another, immunoproteomic approach using 2-DE-MALDI-TOF MS and sera from pigs with ascariasis let to identify 24 immunoreactive proteins [111]. Most of them (23/24) were determined to be related to the survival mechanisms of parasites, involving functions connected with energy production (12 proteins) and redox processes (5 proteins). These results might help to find effective chemotherapeutic targets for porcine ascariasis [111]. Different strategy was used to characterize the protein composition of perienteric fluid (PE), uterine fluid (UF), and total excretory/secretory products (ESP) from this parasite. Chehayeb et al. [112] used SDS-PAGE combined with LC-MS/MS to identify 175, 308, and 274 proteins in ESP, PE, and UF, respectively. The ultra-performance liquid chromatography coupled to nano-electrospray tandem mass spectrometry (UPLC-nanoESI MS/MS) let to identify 268 proteins of extracellular vesicles isolated from *A. suum* by ultracentrifugation. To date, to our knowledge, it is the most comprehensive analysis of protein composition of *A. suum* extracellular vesicles [113].

#### 2.2.4. *Fasciola* spp.

Six different species of plant-borne trematodes are known to affect humans: *Fasciola hepatica*, *F. gigantica*, *Fasciolopsis buski* (Fasciolidae), *Gastrodiscoides hominis* (Gastrodicidae), *Watsonius watsoni*, and *Fischoederus elongates* (Paramphistomidae). Whereas *G. hominis*, *F. buski*, *W. watsoni*, and *F. elongates* are intestinal, the *F. hepatica* and *F. gigantica* are hepatic trematodes. In the present section, we will focus only on the members of Fasciola. Fascioliasis is caused by two species of liver fluke—*F. hepatica* and *F. gigantica*. Fascioliasis affects domestic animals, as well as humans [114]. *F. hepatica* is a cosmopolitan species because it has the ability to infect many different species, and the intermediate snail host has the ability to adapt to various ecological niches [115]. Due to the reduced ability of aquatic snail intermediate hosts to invade new niches, the distribution of *F. gigantica* is more limited, usually to tropical regions of Asia and Africa [116,117]. Outbreaks of human infections are always related to local animal fascioliasis cases [115,117]. It is estimated that between 2.4 and 17 million people are currently infected and 91 million are at risk of infection [118]. Infection is usually spread by various aquatic plants, such as watercress, algae or tortora, on which the metacercaria have settled and are then consumed [114]. Farm management practices and growing aquatic plants in greenhouses have reduced the number of human infection cases in industrialized areas, but still in some developing countries, wild aquatic plants or plants grown in fields, where infected animals can roam freely, they become a threat to humans. In addition, metacercaria can be found floating in the water, so people can get the infection by drinking water [3,116,117]. Clinical symptoms of an acute fascioliasis are abdominal pain, indigestion, weight loss, mild fever. Other gastrointestinal symptoms result from the migration of the young flukes through the liver, which also always results in hepatomegaly [30].

Proteomics has been widely used in the studies on *F. hepatica* excretory–secretory (ES) proteins [119]. Early proteomic studies of *F. hepatica* used radiometabolic markers to distinguish protein profiles at different developmental stages [120,121]. Isoelectric focusing and densitometry were also carried out to characterize the ES proteins secreted by flukes parasitizing diverse mammals [122,123]. Jefferies et al. [124,125] improved this analysis using 2-DE. These studies characterized a range of different glutathione S-transferases, fatty acid binding proteins, superoxide dismutase, peroxiredoxin, and cathepsin L-proteases, which have been further analyzed using more advanced proteomics techniques [126,127,128,129,130,131,132,133,134]. Proteomic methods (2-DE-LC-MS/MS) were also adjusted to describe the mechanism of action of anthelminthic drug triclabendazole on *F. hepatica* [135], as well as to discern protein signatures of this parasite’s susceptible and putatively resistant to triclabendazole [136]. One challenge in the proteomics of *F. hepatica* was to characterize the proteomes of early developmental and migratory stages because of their small size. Moxon et al. proved that eggs have significantly different proteomes from the other life stages of *F. hepatica* [137]. DiMaggio et al. [134], using gel-free proteomic techniques (shotgun proteomics), performed an extensive analysis of the proteins secreted by an adult *F. hepatica* and newly excysted juveniles (NEJ; 48 h post-excystment) and compared these with the somatic proteome of the NEJ 48 h. This study identified 202 proteins in the adult secretory group, 90 proteins in the NEJ 48 h secretory group, and 575 proteins in the NEJ 48 h somatic proteome.

The key to survival in the host environment is the parasite surface that can change quickly to prevent host immune cells from attacking. Proteomic characterization (nanoUPLC–ESI–qTOF–MS and MALDI-TOF-MS) of the *F. hepatica* tegument was also performed [138,139].

#### 2.2.5. *Trypanosoma cruzi*

*Trypanosoma cruzi* (*T. cruzi*) is a species of parasitic euglenoids, which are transmitted to mammals by insects from the subfamily of the Reduviidae, the hematophagous insect triatomine (“assassin bug,” “cone-nose bug,” and “kissing bug”) [140]. The natural habitat of bugs are the burrows and nests of animals. *T. cruzi* reservoirs are armadillos and opossums and, less frequently, rodents, monkeys, dogs, cats, and cattle [141]. Invasive forms for mammals, including humans (metacyclic trypomastigotes), are excreted onto the skin along with feces of bugs during blood sucking. Invasive forms reach the wounds at the site of the bug bites and skin scratches and also through the mucous membranes and the conjunctiva (rubbing the eye with the hand). In the host’s cells, parasites undergo part of the life cycle (from metacyclic trypomastigotes to amastigotes and then trypomastigotes). When infected cells are lysed, parasites are released, then enter subsequent cells and infect them. If the trypomastigote enters the gut of a bug during blood sucking from an infected host, it will complete the life cycle [142]. The disease caused by *T. cruzi* in humans is called Chagas disease (also known as American trypanosomiasis) [143]. Chagas disease has two stages: the acute stage, which develops 1–2 two weeks after the insect bite, and the chronic stage, which develops for many years. Usually asymptomatic is the acute phase [143,144]. In the chronic stage, symptoms include fever, general malaise, headache, hepatomegaly, splenomegaly, and swollen lymph nodes. It is rare for people to have nodules swelling at the site of infection. If it is on the eyelid, it is called “Romaña’s sign” and if it is on the skin, it is called “chagoma” [144]. In rare cases, an infected person will develop a severe acute illness, which may cause life-threatening fluid accumulation around the heart or cause inflammation of the heart or brain. The acute phase usually lasts 4 to 8 weeks and will subside without medication [143].

Proteomics has been widely used in the studies on *T. cruzi.* Large-scale comparative proteomics studies have shown that metacyclic form had the highest number of proteins expressed, followed by amastigotes, epimastigotes, and trypomastigotes [145]. Many other studies showed stage-specific proteins. The shotgun proteomics of the blood trypomastigote stage was used to described the main classes of proteins present in this stage [146]. Comparison of the proteome of blood trypomastigote with that derived from the tissue culture or metacyclic trypomastigote shows that more than 2200 proteins are unique to the blood trypomastigote stage and participate in various cellular processes [146]. Proteomic analysis of the trypomastigote identified more than 1400 proteins, of which nearly 14% are surface proteins anchored by glycophosphatidylinositol (GPI), which might be involved in host cell invasion and immune escape [147]. A study showed that the difference between the protein expressions of the epimastigotes and trypomastigotes was more than 50%. The study also determined that some protein isoforms are involved in metacyclogenesis [148]. Metacyclogenesis, the process of transforming procyclic promastigotes into highly infective metacyclic promastigotes, was also investigated using proteomic tools. Large-scale proteomics research has pointed out major differences in proteins related to oxidative stress, translation, and metabolic pathways related to proteins, lipids and carbohydrates [149]. Quantitation of phosphorylated proteins in the same study showed that there are more than 7000 phosphorylation sites, of which 260 are under different regulation, including some potential drug targets, e.g., sterol biosynthesis enzymes. Next study indicated that during periods of nutritional stress, many proteins are phosphorylated, which may trigger metacyclogenesis [150]. Similarly, the proteomic comparison of the exponential phase and stationary phase of the epimastigotes quantified more than 3000 proteins [151]. The transition from trypomastigotes to amastigotes (amastigogenesis) was described by quantitative proteomics and phosphoproteomics [152,153].

The surface proteomes of *T. cruzi* was also studied. Comparison of surface proteomes at different stages showed that most of the proteins are expressed in more than one stage, but several are specific for particular stage [153]. Another study showed membrane-derived proteins can participate in invasion, adhesion, cell signal transduction, and modify the host’s immune response. A new family of surface membrane proteins called TcSMPs (*T. cruzi* surface membrane proteins) that is conserved among different *T. cruzi* lineages has been characterized [154].

#### 2.2.6. *Trichuris trichiura*

*Trichuris trichiura* (*T. trichiura*) (whipworm) is a nematode that causes trichuriasis in humans. The larvae infects human cecum and colon [155]. Ingestion of embryonated eggs from the external environment can cause infection. After hatching, the larvae emerge from the polar egg and establish an infection in the epithelium of the cecum and colonic Lieberkühn crypts. Following the characteristic four molts, the dioecious adult parasites develop unobstructed (rate depends on the host), mate and hatch unembryonated eggs, which are expelled into the environment through feces. [155]. Trichuriasis is more common in warm climates. If the infected person defecates outdoors, or if untreated human feces is used as fertilizer, the eggs are deposited on the soil and they can mature to the infection stage. Ingestion of these eggs “can happen when hands or fingers that have contaminated dirt on them are put in the mouth or by consuming vegetables or fruits that have not been carefully cooked, washed or peeled” [156].

The proteomic studies were not conducted often on *T. trichiura*. In 1995, the 2D-SDS-PAGE electrophoresis technique was used to describe the ability of excretory/secretory proteins of *T. trichiura* adult worms recovered from the human, to provoke an immune response [157]. Latest proteomic analysis of *T. trichiura* egg extracts using LC-MS/MS revealed potential immunomodulatory and diagnostic targets [158]. Most of all the other proteomic studies, to our knowledge, were conducted on *T. muris*, which has been used for over 50 years as a model for *T. trichiura* [159]. The best-described issue using proteomics methods is the extracellular vesicles’ protein composition. *T. muris* proteins from the vesicular component were analyzed by LC-MS/MS in several studies, and potential immunogenic proteins and new insights into parasite–host communication were described [160,161,162].

### 2.3. Meat-Borne Parasitic Species

Humans get infected by many FBPs by eating uncooked or raw meat infected with development stages of these parasites. Among meat-borne parasitic infection, according the European ranking [36], the most important parasites are: *Toxoplasma gondii* (second/25), *Trichinella spiralis* (third/25), *Trichinella* spp. other than *T. spiralis* (sixth/25), *Taenia solium* (10th/25), *Taenia saginata* (15th/25), and *Sarcocystis* spp. (18th/25). In this section, since there are more proteomics studies from these species, we will only focus on selected parasites.

#### 2.3.1. *Toxoplasma gondii*

*Toxoplasma gondii* (*T. gondii*) is a protozoan parasite that, like *Cryptosporidium* spp., belongs to the phylum Apicomplexa. The parasite has a complex life cycle for which usually domestic cats are the definitive hosts [163]. Intermediate hosts are all warm-blooded animals, including livestock and humans. Infected cats excrete oocysts in the feces. If they are ingested after spore formation, they will infect the intermediate host and develop into rapidly reproducing tachyzoites, which are spread throughout the body [7]. Future mothers are particularly vulnerable, because tachyzoites can cross the placenta and infect the fetus. After tachyzoites are located in the muscle tissue and central nervous system, they transform into tissue cysts (bradyzoites). Food-borne Toxoplasma infection can be obtained by ingesting tissue cysts in uncooked or raw meat, or ingesting oocysts by eating contaminated vegetables or drinking water [7]. In pregnant women, toxoplasmosis is generally considered a serious health problem, which can transmit infection to fetuses or newborns, as well as in people with weakened immune systems. In adults with strong immunity, the infection is usually asymptomatic [3]. Nevertheless, recently published research have shown that almost all cases of ocular toxoplasmosis is caused by acquired diseases, which means that prevention should not only target pregnant women and people with weakened immune functions, but also the general population [164]. Proper meat cooking, as well as freezing for the appropriate time is the best method known to kill toxoplasma cysts [3].

To our knowledge, *T. gondii* is one of the best proteomic-studied FBPs. Before there was a lot of genomic information about the parasite, the Toxoplasma gondii protein was identified by MS, which depends on the use of NCBI and the limited EST database to identify the protein [165,166]. With the development of an annotated genome for *T. gondii*, it was possible to search efficiently in the MS data against thousands of *T. gondii* protein sequences [167]. The tachyzoite has been the main focus of proteomic studies of *T. gondii*, although some data have now been published on the other life cycle stages. Xia et al. [168] published the results of the first multi-platform (1-DE LC-MS/MS, 2-DE LC-MS/MS and MudPIT) proteomic analysis of *Toxoplasma* tachyzoites, identifying nearly one-third of the entire predicted proteome of *T. gondii*. In another study, Dybas et al. [169] using 1-DE LC-MS/MS identified 2477 gene-coding regions with 6438 possible alternative gene predictions—approximately one third of the *T. gondii* proteome. The proteomics investigation found that compared with any known species (including other Apicomplexan), there are 609 unique proteins of Toxoplasma gondii [169]. The proteomic profiles of different genotypes of *T. gondii* tachyzoites using 2-DE difference gel electrophoresis (DIGE) combined with MALDI- TOF MS were also investigated [170]. A different approach, focused on the antigenicity of soluble tachyzoite antigen (STAg), led to the identification of 1227 proteins of *T. gondii* STAg [171]. Through MS analysis, 426 proteins were identified among the 1227 isolated protein spots. A proteogenomic approach has allowed Krishna et al. [172] to reanalyze many published data sets of *T. gondii* and generate new high-throughput MS/MS data sets. Four different techniques (1-DE, 1-DE of soluble and insoluble fractions (1DE SFIF), 2-DE, and MudPIT) were used and obtained samples were analyzed on an LC-MS/MS. The MS data were searched against the protein database assembled from two different sources: the official gene models and predicted gene models supported by RNA-Seq evidences [172]. With use of this proteogenomic approach the identification of 30,494 peptide sequences and 2921 proteins for *T. gondii* was performed. In addition to the tachyzoite stage, oocysts which are highly resistant to the environmental conditions were also studied by global proteomics methods. Fritz et al. [173] have characterized the proteome of the wall and sporocyst/sporozoite fractions of mature, sporulated oocysts using the 1-DE LC-MS/MS approach. A total of 1021 Toxoplasma proteins were identified in the sporocyst/sporozoite fraction and 226 proteins were identified in the oocyst wall part. Importantly, 172 proteins were identified as not reported in other *Toxoplasma* proteomic evaluations. Moreover, the application of isotope tags for relative and absolute quantification (iTRAQ) coupled with 2-DE LC–MS/MS to investigate the proteome of oocysts during sporulation, let to describe 2095 proteins where 587 were identified as differentially regulated (sporulated and non-sporulated oocysts) [174].

Excretory-secretory proteins were also investigated in *T. gondii*. Zhou et al. [175] have applied proteomics techniques to analyze a large number of freely released Toxoplasma secretory proteins by using 2-DE and MudPIT. Another group using LC-MS/MS identified excretory-secretory proteins from the RH strain of *T. gondii* [176]. A total of 34 proteins were identified and their abundance was estimated by spectral counting method. Among them, eight microparticle proteins (MICs), two species of rhoptry proteins (ROPs) and six dense granular proteins (GRAs) were identified [176].

The most comprehensive description of the proteomic organization of a *T. gondii* cell (tachyzoite) was recently presented by Barylyuk et al. [177] by applying relatively new proteomic method of subcellular localization of thousands of proteins per experiment by isotope tagging (hyperLOPIT). The hyperLOPIT method utilizes a unique abundance distribution map, which is formed during the organelles and subcellular structures biochemical fractionation, e.g., density gradient centrifugation. Proteins showing similar abundance distribution characteristics through these fractions are assigned to proper subcellular structures [178,179]. In each of three experiment replicates, Barylyuk et al. identified over 4100 proteins across all 10 fractions representing subcellular compartments of *T. gondii* trachyzoite. In addition, these three data sets have a total of 3832 proteins, which can provide complete abundance distribution overview information of 30 fractions. Using the hyperLOPIT approach, Barylyuk et al. assigned thousands of proteins to their subcellular niches [177].

#### 2.3.2. *Trichinella* spp.

Trichinella genus is one of the most widespread group of parasitic nematodes in the world. With the exception of Antarctica, Trichinella infections have been detected in domestic and wild animals on all continents [3]. Not long ago, all Trichinella infections that occurred in animals and humans were attributed to *T. spiralis*. Nowadays, eight species and four genotypes within two clades (encapsulated and non-encapsulated) are recognized in this genus [4,180]. Trichinellosis is caused by *Trichinella* larvae that are encysted in muscle tissue of domestic or wild animal meat. The domestic pig is considered as the most important source of human infection worldwide. However, in the past few decades, wild boar and horse meat have played similar role [181]. Infection is characterized by fever, diarrhea, periorbital oedema, and myalgia. Many severe complications like myocarditis, thromboembolic disease, and encephalitis may occur [15,181]. Europe has issued official regulations, which provide for the control of Trichinella in meat to improve consumers safety [9].

Therefore, *Trichinella* spp. is not just a hazard to public health, but also an economic problem in porcine animal production. Due that many scientific groups are working on methods to control and elimination of this parasite from the food chain. Proteomics methods are also used to help solve this problem. 

Liu et al., using iTRAQ method, has described differentially regulated proteins in the three stages of *T. spiralis*—adult (Ad), muscle larvae (ML), and newborn larvae (NBL) [182]. A total of 4691 proteins were identified in all the stages, of which 1067 were differentially regulated. Different work performed on *T. spiralis* used label-free LC–MS/MS to determine the proteome differences between *T. spiralis* ML and intestinal infective larvae at the molting stage [183]. A total of 2885 proteins were identified, of which 323 were differentially regulated. These proteins were involved in regulation of cuticle synthesis, remodeling and degradation, and hormonal regulation of molting. In another study conducted on *T. britovi* (the second most common species), somatic extracts obtained from ML and Ad were separated using 2-DE coupled with immunoblot analysis. Then, the protein spots were identified by LC-MS/MS [184]. A total of 272 proteins were identified in the proteome of *T. britovi* Ad, and 261 in ML. Somatic cell extracts of Ad and ML were specifically recognized by *T. britovi*-infected swine serum 10 days after infection, with a total of 70 prominent proteins [184]. Proteomic analyses of species specific antigens were also performed with the use of MALDI-TOF and MALDI-TOF/TOF [185,186]. Potentially immunogenic proteins of the encapsulated (*T. spiralis*) and non-encapsulated (*T. pseudospiralis*, *T. papuae*) species were also investigated [187], and such proteins were identified by LC-MS/MS. Then, their possible functions were determined using gene ontology analysis. Host–parasite interactions were also analyzed by investigation of surface and excretory-secretory proteins of *Trichinella* spp. The surface proteins of *T. spiralis* muscle larvae were detected by 2-DE and MS. The 2-DE analysis detected about 33 protein spots, of which 14 were identified in the serum of mice infected with *T. spiralis*, and 12 were successfully identified by MALDI-TOF/TOF-MS [188]. The same group, using shotgun LC-MS/MS, performed comparative proteomic analysis and described surface protein profiles of ML and intestinal infective larvae [189]. A total of 41 proteins were shared by both stages, while ML had 85 and intestinal infectious larvae had 113 stage-specific proteins. Certain proteins (for example, putative onchocystatin) were involved in host-parasite interactions. Excretory–secretory proteins, as the most important products of host–parasite interaction, were investigated in the latest studies on *T. spiralis*, *T. pseudospiralis* and *T. britovi* [190,191,192].

#### 2.3.3. *Taenia* spp.

The terms “cysticercosis” and “taeniosis” respectively refer to foodborne zoonotic infections with larval and adult tapeworms of the genus *Taenia*. The larvae of these tapeworms are meat-borne (beef or pork) and the adult stage is an obligate parasite of the human intestine [193]. *T. solium* (pork) and *T. saginata* (beef) are the most important causes of taeniosis in Europe [36]. Within the European Union, certain countries can acquire *T. solium* infection locally. There have been reports of pig infections in Hungary, Lithuania, Austria, Estonia, Romania, and Poland [194], while there were only sporadic imported cases in other countries. Humans obtain tapeworms by eating raw or undercooked infected meat. Among these tapeworms, *T. solium* is exclusive because the cysticercus stage can also infect humans directly. Human cysticercosis is acquired by accidental ingestion of *T. solium* cysticerci excreted in host feces. In humans, cysticerci may lodge in the brain and cause neurocysticercosis [195]. Taeniasis in humans is of minor clinical significance; usually, asymptomatic or symptoms are mild and non-specific (abdominal pain, weight loss, nausea, diarrhea or constipation and itching cause d by proglottids, which might be passed through the anus) [15,193]. Nevertheless, cysticercosis does have major clinical significance. Intracranial hypertension and epilepsy are the most common clinical manifestations [194].

So far, to our knowledge, proteomics has described the fallowing main *Taenia* spp. features: total protein composition of cysticerci of *T. solium* by 2-DE [196]; in *T. solium,* using LC-MS/MS, a set of oncosphere proteins involved in gut penetration and immune evasion machineries in adhesion [197]; candidate antigens through immunoproteomics [198,199,200,201]; *T. solium* cysts proteomes obtained from different host tissues [202,203]; saline vesicular extract proteins of *T. solium* [204]; and *T. solium* excretory-secretory proteome [205].

#### 2.3.4. *Sarcocystis* spp.

In pigs, three species of *Sarcocystis* were found: *S. miescheriana*, *S. porcifelis*, and *S. suihominis.* However, only *S. suihominis* can cause human infections when eating raw pork [206]. *S. suihominis* has an obligatory two-host life cycle. Sporocysts are shed in the feces of humans or chimpanzees, rhesus and cynomolgus monkeys (definitive host), and pigs (the intermediate host). In pigs, parasites are encapsulated in muscle tissue, but usually do not cause pathological changes or symptoms [193,207]. Infection can be asymptomatic or symptomatic (nausea, loss of appetite, stomach pain, vomiting, diarrhea, difficulty in breathing, and rapid pulse) [193]. Sarcosporidiosis is a self-limiting infection and treatment is not known.

Until now, to our knowledge, there is no published report at the proteomic analysis of *S. suihominis.*

### 2.4. Seafood-Borne Parasitic Species

Fish meat can be infected by a variety of parasites, which can cause human infections when eaten raw or undercooked. Additionally, various species of shellfish (mollusks and crustaceans) can be consumed by people when infected by different stages of many parasites. In addition, in many parts of the world, the term “seafood” has been extended to freshwater organisms consumed by humans, so all edible aquatic organisms can be called “seafood”, including aquatic plants. Due that, in this work and according to the ranking prioritizing foodborne parasites in Europe, we describe not only sea-species parasites but also freshwater-parasitic species. Among seafood-borne parasitic infection, according the European ranking [36], the most important parasites are Anisakidae (seventh/25), Opisthorchiidae (11th/25), *Angiostrongylus cantonesis* (13th/25), *Diphyllobothrium* spp. (16th/25), *Paragonimus* spp. (23rd/25), and Heterophyidae (24th/25). Due to emerging number of Anisakidae infections in Europe and strong allergic reaction to, e.g., *Anisakis simplex* s.s., we decided to discuss the Anisakidae family in a separate section.

#### 2.4.1. Opisthorchiidae

Opisthorchiasis is a trematode infection caused by species of the family Opisthorchiidae, specifically, *Opisthorchis viverrini* and *O. felineus* [30]. It is calculated that around 10 million people have been infected with *O. viverrini* [208], and 67 million are at risk of infection [209]. The freshwater snail is a first intermediate host of *O. viverrini*, while the second intermediate hosts include several freshwater cyprinid fish species [210]. Freshwater fish dishes infected with metacercariae have are the main source of infection of this parasites to humans [211]. Human opisthorchiasis is typically asymptomatic and therefore results in chronic inflammatory disease; this chronic inflammation can develop into the cholangiocarcinoma [212]. Thus, *O. viverrini* has been classified by the International Agency for Research on Cancer as a group 1 carcinogen. The only way to reduce the percent of cholangiocarcinoma cases, where the causative agent was *O. viverrini*, is to reduce the prevalence of opisthorchiasis through the use of praziquantel—an anthelminthic drug [212]. Unfortunately, this drug is at risk of resistance, and studies performed on *O. viverrini* could help develop efficient methods to reduce the prevalence of opisthorchiasis and induced by O*. viverrini* cholangiocarcinoma [212].

Comparative 2-DE analysis was used to highlight proteins that are significantly modulated during the maturation stage of *O. viverrini.* The differentially regulated proteins in the juvenile/adult form of the parasite are thought to be important for survival and pathogenesis. Compared with the one-week-old juvenile fluke, 35 protein spots in four-week-old adults were differentially regulated. [213]. Moreover, using proteomics (QTRAP MS/MS) Mulvenna et al. [214] characterized 300 proteins from the *O. viverrini* excretory-secretory products. In addition, more than 160 tegumental proteins were identified using sequential solubilization of isolated teguments, and some of them were located on the surface membrane of the tegument by localizing with fluorescence microscopy. The several proteins functions are still unknown [214]. Studies on proteomes of intermediate hosts of *O. viverrini* were also conducted. Proteomic profile using iTRAQ labelling technology of *Bithynia siamensis goniomphalos* snails upon infection with the *O. viverrini* was characterized [215]. This study indicates that motor proteins, and stress-related proteins are greatly upregulated after infection. In addition, the expression level of peroxiredoxins was reduced in infected *Bithynia*. Using sequential window acquisition of all theoretical spectra mass spectrometry (SWATH-MS), the protein composition of the hemolymph of *B. siamensis goniomphalos* infected with *O. viverrini* was described. The analysis revealed the presence of 242 and 362 proteins in the plasma and hemocytes, respectively [216]. Among them, the 117 and 145 proteins showed significant differences after opisthorchiasis in plasma and hemocytes, respectively. Suwannatrai et al. [216], among proteins with significantly different expression, found proteins strongly associated with immune response and proteins belonging to the structural and motor categories. 

Although there are still few proteomics studies on *O. viverrini* and its hosts, many of the discovered proteins have become potential candidates for diagnostic biomarkers or new drug development.

#### 2.4.2. *Angiostrongylus cantonesis*

*Angiostrongylus cantonensis* (*A. cantonensis*) is a parasitic nematode that occasionally causes angiostrongyliasis in humans. Its main clinical manifestation is eosinophilic meningitis [217]. Human infections are acquired by ingestion of raw or undercooked snails or slugs, paratenic hosts such as prawns, or contaminated vegetables that contain the infective larvae. After swallowing, the infective larvae are digested from these carriers and invade the intestinal tissues, causing human enteritis, and then pass through the liver. When the worm moves through the lungs, cough, rhinorrhea, sore throat, discomfort and fever occur. In about 14 days, the larvae reach the central nervous system, followed by eosinophilic meningitis and eosinophilia [217,218]. In many patients, the larvae can also move to the eyes and cause ocular angiostrongyliasis, accompanied by visual disturbances, such as diplopia or strabismus [219,220]. Detection of *A. cantonensis* in cerebrospinal fluid or the ocular chamber confirms the disease in humans. However, the percentage of confirmed cases is very low. The history of eating intermediate or paratenic hosts in medical interview is essential for the diagnosis [217]. The combination of corticosteroids and anthelmintics has been commonly used to treat this disease [217].

*A. cantonensis* has been widely studied using proteomic methods. The protein expression profiles of the parasite’s infective third and pathogenic five stage larvae were compared by proteomics technology [221]. Isolated protein samples were separated by 2-DE, and analyzed by MALDI-TOF MS. Of the 100 protein spots identified, 33 were from L3, while 67 from L5 and 63 had known identities, and 37 were hypothetical proteins. There were 15 spots of stress proteins, and heat shock protein 60 was the most frequently found stress proteins in L5. Moreover, four protein spots were identified in the serum of the rat host by Western blotting. These changes may reflect the development of L3 from the poikilothermic snails to L5 in the homoeothermic rats [221]. The proteomes of different life stages of *A. cantonensis* were studied more widely by Huang et al. [222], who extracted soluble proteins from various stages of the *A. cantonensis* life cycle (female adults, male adults, the fifth-stage female larvae, the fifth-stage male larvae, and third-stage larvae), separated those proteins using 2D-DIGE and analyzed the gel images. Proteomics analysis yielded a total of 183 different protein spots. Through MALDI-TOF MS/MS, 37 proteins were found with a high confidence score (around 95%). Among them, 29 proteins were identified as cytoskeleton-related proteins and functional proteins [222]. The latest study aimed to identify and characterize the excretory-secretory protein profile of *A. cantonensis* adult larvae [223]. A total of 51 spots were identified using 2-DE. Then, approximately 254 proteins were identified by LC-MS/MS and further classified according to their biological functions. Finally, in the pool of excretory-secretory products of *A. cantonensis* the immunoreactive proteins were identified, including proteins like, disulphide isomerase, putative aspartic protease or annexin [223].

All this information may be useful for discovering biomarkers to diagnose highly dangerous angiostrongyliasis.

#### 2.4.3. *Diphyllobothrium* spp.

Diphyllobothriasis is caused by flatworms of the genus *Diphyllobothrium*, and is acquired by ingestion of larval stages (plerocercoids) present in raw or undercooked fish [224]. *D. latum* is the main species infecting humans. Worms usually reside in the ileum and rarely attach to the bile ducts. In Switzerland, Italy, and France around lakes, reports of diphyllobothriasis have increased, where raw or undercooked perch was consumed. In some countries, previously considered disease-free (Austria, Czech Republic, Belgium, Netherlands and Spain), few cases have been reported, probably related to the consumption of imported raw fish [225]. Although most *Diphyllobothrium* species are large (2–15 m) and can have a mechanical effect on the host, infections are often asymptomatic. About 20% of people experience diarrhea, discomfort and abdominal pain. Other symptoms may also occur, such as fatigue, constipation, pernicious anemia, headache, and allergic reactions. Although large-scale infection is not common, it may cause intestinal obstruction, and the migrating segments can cause cholecystitis or cholangitis [224]. A single dose of praziquantel is highly effective against diphyllobothriasis [225].

Despite the high prevalence of this disease (about 20 million people infected worldwide), to our knowledge, *D. latum* proteome has not been described.

#### 2.4.4. *Paragonimus* spp.

There are about 15 species of *Paragonimus* known to infect humans, while *P. westermani* is the most common etiological agent of human paragonimiasis in Europe [226]. After ingesting raw or undercooked freshwater crustaceans (such as crabs, shrimps or crayfish), humans or other final hosts (carnivores) can become infected. The metacercariae excyst in the small intestine and passes through the intestinal wall into the abdominal cavity before it migrates through the sub-peritoneal tissues, and finally enters the lung where maturation occurs. Eggs of adult individuals, which are coughed up and ejected by spitting with the sputum or swallowed and passed in the feces, hatch, and miracidia invade freshwater snails. Then the cercariae emerge, and crustacea consuming may get infected by consuming it directly or eating infected snails containing the fully developed cercariae [30]. The presence of paragonimiasis can cause bleeding, inflammation, lung parenchymal necrosis and fibrotic cysts. In lung paragonimiasis, the most obvious symptom is chronic cough, accompanied by brown and bloody pneumonia-like sputum [30,226].

Despite the importance of the disease, little information about the proteomics of *Paragonimus* spp. can be found. The only analyzed excretory–secretory products of adult *P. westermani* using 2-DE coupled to MS [227]. In this study 25 different proteins were identified, some of which are highly representative, such as cysteine proteases. In addition, three previously unknown cysteine proteases were also identified by MALDI-TOF/TOF MS, and most of them are reactive to serum from patients with paragonimiasis. Park et al. [228] suggested that a new drug for paragonimiasis could be designed, focusing on exploring inhibitors of cysteine proteases.

#### 2.4.5. Heterophyidae

In humans, heterophyidiasis and metagonimiasis is associated mainly with species of *Heterophyes* or *Metagonimus*, respectively. Those diseases are the best-known associated with heterophyid parasitism [229]. Humans can get infected usually by eating raw, undercooked or under-processed fish [230]. The two most widespread species of heterophyids are *H. heterophyes* and *M. yokogawai*. There are evidences in European countries of infections caused by *H. heterophyes* (Spain, Italy, Greece, Turkey) and *M. yokoagwai* (Bulgaria, the Czech Republic, Romania, Serbia, Spain, and Ukraine). Intriguing, there have been no reports of *M. yokoagwai* infecting humans [229].

The parasitic hosts of *H. heterophyes* include dogs, cats, pigs, fish-eating birds, and other fish-eating mammals. Adult worms live in the small intestine of vertebrate hosts and the gastropods from the genus *Semisulcospira* are the earliest intermediate hosts, where cyprinid fish are mainly a second intermediate host [230]. Most heterophyids infections have no clinical consequences, but severe ones are causing gastro-intestinal problems [230].

The genome of *M. yokogawai* has not been sequenced. To our knowledge, there is no information about proteomics of *M. yokogawai*, and of *H. heterophyes*.

## 3. Targeted Approach—Proteomics Methods Proposed to Use for Detection of Selected FBPs in Food

The one of the major worldwide concern is food safety [231]. Foods contaminated by a range of FBPs is a serious issue causing economic losses in the food sector because it undermines consumer confidence and lowers the demand for potentially infected food products [7,15]. There is a need for technologies that can detect pathogens quickly and early to ensure enhanced food safety. To date, no guidelines or microbiological criteria exist for most FBPs in food products. Advanced proteomic-based methods, like MS, have a great potential for FBPs identification in food. However, detection methods currently available for the selected FBPs are mostly established on standard parasitological approaches (e.g., FBP detection in food product sample by visual examination or microscopy) or on by the polymerase chain reaction (PCR) and enzyme-linked immunosorbent assay (ELISA) [15,55,232,233]. Moreover, most of the conventional proteomic techniques such as ion exchange chromatography, size exclusion chromatography, affinity chromatography, ELISA, western blotting SDS-PAGE, 2-DE, and 2DE-DIGE are used for detection of fungi and bacteria [234,235,236]. MALDI-TOF MS, surface-enhanced laser desorption ionization time of flight mass spectrometry (SELDI-TOF MS), LC-MS/MS, isotope-coded affinity tags (ICAT), and iTRAQ are the central among current proteomics. The discovery proteomic workflow used for identification of biomarkers of parasitic infections is essential for diagnostic purposes and new treatment inventions. Biomarkers can be either proteins of the parasite itself or host proteins responding to infection. Then, the targeted proteomics could be used to search with high precision, sensitivity and reproducibility the peptide biomarkers selected in the discovery phase in patient biological fluids or in food products [17,237]. Although identification of protein targets has been done in many FBPs, the proteomic methods to detect them are still rare. SELDI is one of the most used in the studies published about parasitic diseases. This proteomic method has been applied to investigate the serum biomarkers of African trypanosomiasis [238], fascioliasis [239], cysticercosis [240], and Chagas disease [241]. These studies have focused on identifying a “proteomic fingerprint” in infected people’s/animals’ serum—a unique configuration of parasite proteins that indicate a specific pathophysiological state. Even so, these methods are not widely used. In our opinion, there is a colossal need to diagnose foodborne infections using new methodologies, such as MS and biomarkers detection, according to their extreme specificity and possible increase of the detection rate [242].

Another factor that has attracted increasing attention to FBPs is the increasing demand for protein-rich foods such as fish. [243]. Thus, seafood-borne parasitic infections, such as those caused by Heterophyidae, Opisthorchiidae, and Anisakidae, are emerging ones, and development of advanced and more accurate methods for identification, monitoring, and assessing of FBPs during production, processing, and storage should be now worldwide concern.

Therefore, in this part, we focused on the brief description of the proteomic methods proposed to use for Anisakidae detection in food products.

### Anisakidae

The consumption of raw or unprocessed fish infected with cosmopolitan nematodes belonging to the Anisakidae family may lead to anisakidosis [244]. Known human-infecting Anisakids species include members of the *Anisakis simplex* complex (*A. simplex* sensu stricto, *A. pegreffii*, *A. berlandi*), the *Pseudoterranova decipiens* complex (*P. decipiens* sensu stricto, *P. azarasi*, *P. cattani*, and others), and the *Contracecum osculatum* [244]. Among them, *A. simplex* is the most commonly involved in human infections, and the disease caused by the *Anisakis* genus is called anisakiasis [245,246]. The life cycle of *A. simplex* is complex and involves four larval stages parasitizing several intermediate and paratenic hosts (fish, cephalopods, and crustaceans) and the adult stage parasitizing marine mammals (seals, dolphins, and whales). Humans can be accidentally infected by eating raw or undercooked fish or seafood contaminated by third-stage (infective) development stage [244,247].

The ingestion of viable larvae might lead to gastrointestinal symptoms (abdominal pain, nausea, vomiting, diarrhea), which may be associated with mild to severe allergic reactions, and the clinical symptoms most often are such as rhinitis, urticaria, and, in worst cases, anaphylactic shock [245,246]. Although, cooking (or freezing) is expected to kill the parasites, it might not decrease its allergenicity, because *A. simplex* allergens have high heat and frost resistance; and sensitization may occur after consumption [247].

Proteomic studies on *A. simplex* were reviewed meticulously and accurately by D’Amelio et al. [248]. In brief, using MALDI-TOF/TOF analysis, differentially expressed proteins for *A. simplex* s.s., *A. pegreffii*, and their hybrid were described, and potential new allergens were identified. In a similar study, Fæste et al. [249] characterized, using sera from *A. simplex*-sensitized patients, potential allergens. Additionally, biomarker (peptides) for relevant *A. simplex* proteins were described. Most of all, *A. simplex* allergens have been identified and characterized [248]. Recently, for the first time, the global proteome of the third and fourth stage larvae of *A. simplex* was analyzed using quantitative proteomics based on tandem mass tag (TMT) [31]. In addition, the response to the invasive larvae of *A. simplex* s.s. to ivermectin (anthelminthic drug) was also evaluated using TMT-based methodology [250].

The discovery of potential allergens has prompted scientists to use a targeted approach and develop methodologies for detection of Anisakids in food products. Lately, the method for detection of *A. simplex* allergens in fresh, frozen, and cooked fish meat was proposed with the use of immunoglobulin G (IgG) immunoblotting [251]. Recently, many Anisakids proteins have been identified through LC-MS/MS-based proteomics, which laid the foundation for the rise of detection methods of *A. simplex* in fish. Fæste et al. [252] have shown by ELISA, immunostaining, and MS, proteins of *A. simplex* in salmon meant for use in sushi and other fish products on the Norwegian market. The same group proposed two more methodologies for *A. simplex* protein detection in fish [253]. Both were based on multiple reaction monitoring (MRM)-MS/MS, which is applied to quantify previously identified target peptides by measuring specific precursor-to-product ion transitions. Both proposed methodologies, the label-free semi-quantitative nLC-nESI-Orbitrap-MS/MS and the heavy peptide-applying absolute-quantitative (AQUA) LC-TripleQ-MS/MS use unique reporter peptides derived from Anisakids hemoglobin and SXP/RAL-2 protein as analytes. 

Recently, the analysis performed by Carrera et al. [254] showed possible practical use of peptide biomarkers in food industry. The discovery phase was based on the isolation of heat-stable proteins of *A. simplex*, *P. krabbei*, and *P. decipiens*, then the use of accelerated in-solution trypsin digestions under an ultrasonic field provided by high-intensity focused ultrasound (HIFU) and the monitoring of several peptide biomarkers by parallel reaction monitoring (PRM) mass spectrometry in a linear ion trap mass spectrometer. The target detection step showed the same proportional relationships between the proposed peptide biomarkers that spiked in hake protein extracts, like those of the buffer diluted sample, which confirms the effectiveness of the PRM method in real fish samples. This method can quickly detect Anisakids in less than 2 h [254], and if it is made a part of a control protocol defined by food safety authorities, it may facilitate testing and thus increase consumer safety.

## 4. Conclusions and Future Directions

These days the Proteomics provides a great tool in both basic and applied parasitology. Proteomics methods, like SDS-PAGE, 2-DE, and LC-MS/MS combined with bioinformatic tools, have become common in modern helminth parasitology research. These methods have the potential to identify differentially regulated proteins in diverse parasite development stages or in response to drugs, as well as to describe the composition of parasitic extracellular vesicles. The discovery of new parasite proteins, consequently, might help to find candidates for the parasites’ detection, modern therapies, and vaccines. Although proteomics has broadened the parasitologists view on the parasites’ physiology and parasite–host interactions and crosstalk, little is known about many foodborne parasites and diseases caused by them, including heterophyiasis (*H. heterophyes*), diphyllobothriasis (*D. latum*), sarcosporidiosis (*Sarcocystis* spp.), or paragonimiasis (*P. westermani*). Despite this, great attempts have been made to point out and highlight the importance and benefits of using advanced proteomics methods for the detection of FBPs in food.

Nevertheless, the use of proteomic tools, including software for equipment, databases, and the requirement of skilled personnel, significantly increases costs and therefore limits their wider use. Currently, the potential of proteomics has been used as a finding tool for novel biomarkers, which can then be integrated into uncomplicated diagnostic methods based on inexpensive technologies such as antigen detection in immunochromatographic analysis and other biosensors. Prospective strategies should concentrate on developing proteomic methodologies that are accessible to analytical laboratories or even to the laboratories of food processing plants, with reduced expenses and time-to-result. Furthermore, developed methods should be appropriate for a broad spectrum of different food types and parasite development stages contaminating them, because single methods or biomarkers might not be suitable for the detection of different FBPs stages in a variety of types of food.

Parasite proteins discovery might help developing methodologies for the rapid detection of these contaminants in food products and fill the gap in fields of animal production, agriculture, food processing, and storage, thus benefiting human health. We hope that proteomic methods will be key in opening the door into the increases of detection rate of foodborne parasites in foods, as well as into the reduction of the prevalence of FBPs caused diseases.

## Figures and Tables

**Table 1:** Ranking of foodborne parasites in terms of their importance and risk for european countries according to the criteria of the World Health Organization (WHO) and Food and Agriculture Organization of the United Nations (FAO). To see detailed multi-criteria decision analyses, see reference [36].

Rank	Foodborne Parasites	Infective Life Stage	Transmission Route
1	*Echinococcus multiocularis*	Eggs	soilborne
2	*Toxoplasma gondii*	Fecal oocyst or tissue cyst (bradyzoites)	soil- and meatborne
3	*Trichinella spiralis*	Larvae in a nurse cell	meatborne
4	*Echinococcus granulosus*	Eggs	soilborne
5	*Cryptosporidium* spp.	Oocysts	waterborne
6	*Trichinella* spp. other than *T. spiralis*	Larvae	meatborne
7	Anisakidae	Larvae	seafood-borne
8	*Giardia lamblia*	Cysts	waterborne
9	*Toxocara* spp.	Eggs	soilborne
10	*Taenia solium*	Eggs/Cysticerci	meatborne
11	Opisthorchiidae	Metacercariae	seafood-borne
12	*Ascaris* spp.	Fertilized eggs	soilborne
13	*Angiostrongylus cantonesis*	Larvae	seafood-borne
14	*Entamoeba histolytica*	Cysts	waterborne
15	*Taenia saginata*	Eggs/Cysticerci	meatborne
16	*Diphyllobothrium* spp.	Plerocercoid larvae	seafood-borne
17	*Fasciola* spp.	Metacercariae	plantborne
18	*Sarcocystis* spp.	Cysts with bradyzoites	meatborne
19	*Trypanosoma cruzi*	Metacyclic trypomastigotes	soilborne
20	*Balantidium coli*	Cysts	soil- and waterborne
21	*Cyclospora cayetanensis*	Sporulated oocysts	waterborne
22	*Trichuris trichiura*	Eggs	soilborne
23	*Paragonimus* spp.	Metacercariae	seafood-borne
24	Heterophyidae	Metacercariae	seafood-borne
25	*Spirometra* spp.	Pro-/Plerocercoid larvae	water- and meatborne

**Table 2:** Selected FBPs systematics. Taxonomy has been adopted from the National Center for Biotechnology Information (https://www.ncbi.nlm.nih.gov/taxonomy). For the purposes of this work, slected species of FBPs from three main systematic groups (Platyhelminthes, Namatoda, and Protozoa) were listed. Additionally, the common name of the parasite was added if applicable, the name of the disase caused by each FBP, as well as the human organs where the parasites occure. Legend: *—phylum, ^—class, ”— clade, ’—order.

Systematics				Species/Caused Disease	Common Name/Human Organ Where Occures
**Flatworm infection**	Platyhelminthes *	Rhabditophora ^	Trematoda ” Plagiorchiida ’	*Fasciola hepatica/* *F. gigantica* Fasciolosis	Common liver fluke; liver
				*Opisthorchis viverrini*/ *O. felineus* Opisthorchiasis	Southeast Asian/Cat liver fluke; liver
				*Paragonimus westermani* Paragonimiasis	Oriental lung fluke; lung
				*Heterophyidae* Heterophyiasis	- ; small intestine
		Cestoda ^	Cyclophyllidea ’	*Echinococcus granulosus*/ *E. multilocularis* Echinococcosis	Dog tapeworm/Hydatid worm; liver and other organs
				*Taenia saginata*/*T. solium* Taeniasis/Cysticercosis	Beef/Pork tapeworm; small intestine
			Pseudophyllidea ’	*Diphyllobothrium latum* Diphyllobothriasis	Broad fish tapeworm; small intestine
				*Spirometra* spp. Sparganosis	- ; subcutaneous tissues or muscle
**Roundworm infection**	Nematoda *	Chromadorea ^	Rhabditidia ’	*Angiostrongylus cantonensis* Angiostrongyliasis	Rat lungworm; brain and nervous system
			Ascaridida ’	*Ascaris lumbricoides* Ascariasis	Large roundworm; small intestine
				*Anisakis simplex* s.s. */A. pegreffii* Anisakiasis	Herring worm; gastrointestinal tract
				*Toxocara canis*/*T. cati* Visceral larva migrans/Toxocariasis	Dog/feline roundworm; eye, liver, lungs etc.
		Enoplea ^	Trichocephalida ’	*Trichinella spiralis* Trichinosis	Trichna worm; intestine, muscle and sometimes other organs
				*Trichuris trichiura* Trichuriasis	Whipworm; large intestine
**Protozoan infection**	Apicomplexa *		Eucoccidiorida ’	*Toxoplasma gondii* Toxoplasmosis	- ; brain, eye, lungs, heart, muscle etc.
				*Cryptosporidium parvum* Cryptosporidiosis	- ; intestinal tract
				*Sarcocystis* spp. Sarcocystosis	- ; blood vessels, muscles, intestine
				*Cyclospora cayetanensis* Cyclosporiasis	- ; stomach, small intestine
	Metamonada *		Diplomonadida ’	*Giardia lamblia* Giardiasis	- ; small intestine
	Amoebozoa *		Amoebida ’	*Entamoeba histolytica* Amoebiasis	- ; large intestine and other organs
	Euglenozoa *		Kinetoplastida ’	*Trypanosoma cruzi* Chagas disease	- ; heart, oesophagus, colon, nervous system
	Ciliophora *		Heterotrichida ’	*Balantidium coli* Balantidiasis	- ; cecum and colon
